# Obstructive jaundice due to ampullary metastasis of renal cell carcinoma

**DOI:** 10.1186/1477-7819-11-262

**Published:** 2013-10-07

**Authors:** Andreas Karakatsanis, Antonios Vezakis, Georgios Fragulidis, Chryssa Staikou, Eleni E Carvounis, Andreas Polydorou

**Affiliations:** 1 2nd Department of Surgery, Aretaieion Hospital, Medical School, National and Kapodistrian, University of Athens, Athens, Greece; 2 1st Department of Anesthesiology, Aretaieion Hospital, Medical School, National and Kapodistrian, University of Athens, Athens, Greece; 3 Department of Pathology, Aretaieion Hospital, Medical School, National and Kapodistrian, University of Athens, Athens, Greece

## Abstract

Renal cell carcinoma is often characterized by the presence of metachronous metastases in unusual sites. The presence of isolated metastases is treated with surgical excision with good anticipated results. On the other hand, systemic chemotherapy is administered in the context of metastatic spread, usually sunitib or sorafenib. In such cases, however, the presence of symptomatic foci calls for minimal intervention.

We present a case of a 77-year-old patient who presented with obstructive jaundice due to an ampullary mass. Endoscopic excision and biopsy set the diagnosis of metastatic renal cell carcinoma. Consequently, imaging studies revealed the presence of multiple foci in the lungs and bone. Therefore, pancreatoduodenectomy was excluded and the patient underwent endoscopic ampullectomy and was set to oral sunitinib. Interestingly, despite generalized spread, local control was achieved until the patient succumbed to carcinomatosis.

Painless obstructive jaundice in a patient with history of renal cancer and negative computed tomography scanning for pancreatic or other causes of obstruction should alert for prompt investigation for an ampullary metastasis.

## Background

Obstructive jaundice is one of the most typical clinical signs caused by inflammation, gallstones or tumors of the periampullary region. Painless and progressive rise of serum bilirubin, however, is mostly attributed to tumorigenic entities, rather than inflammatory processes.

## Case presentation

**Figure 1:**
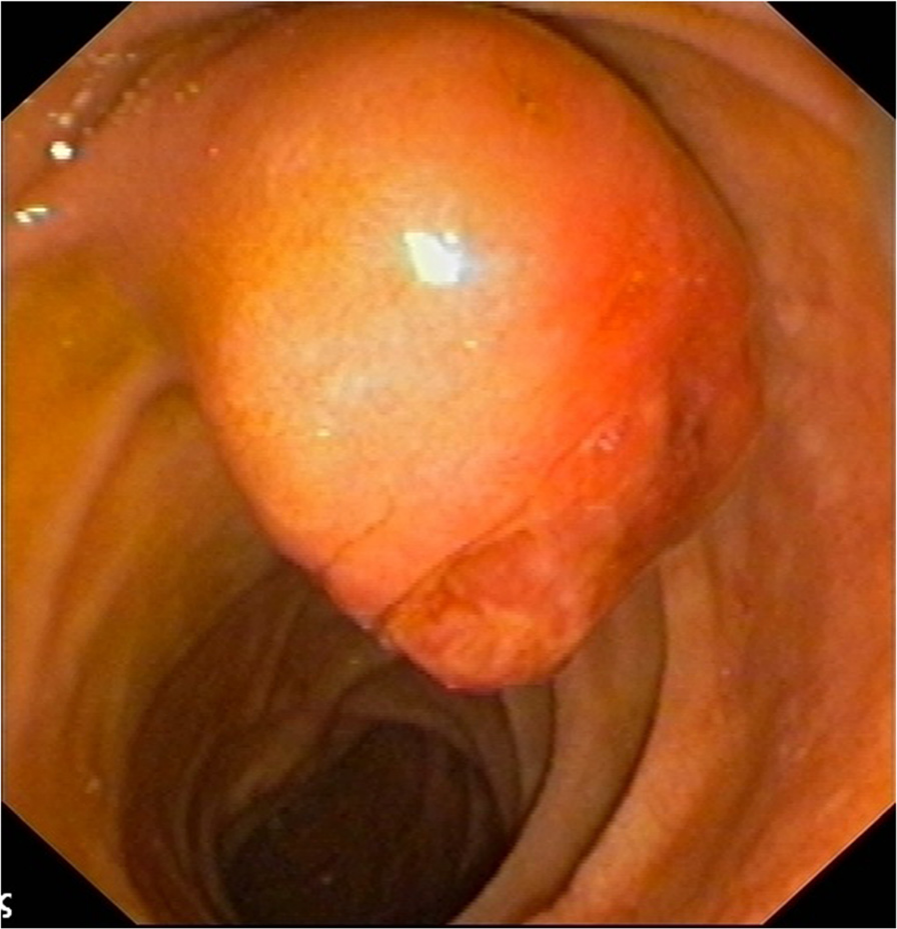
The ampullary mass depicted in endoscopy.

**Figure 2:**
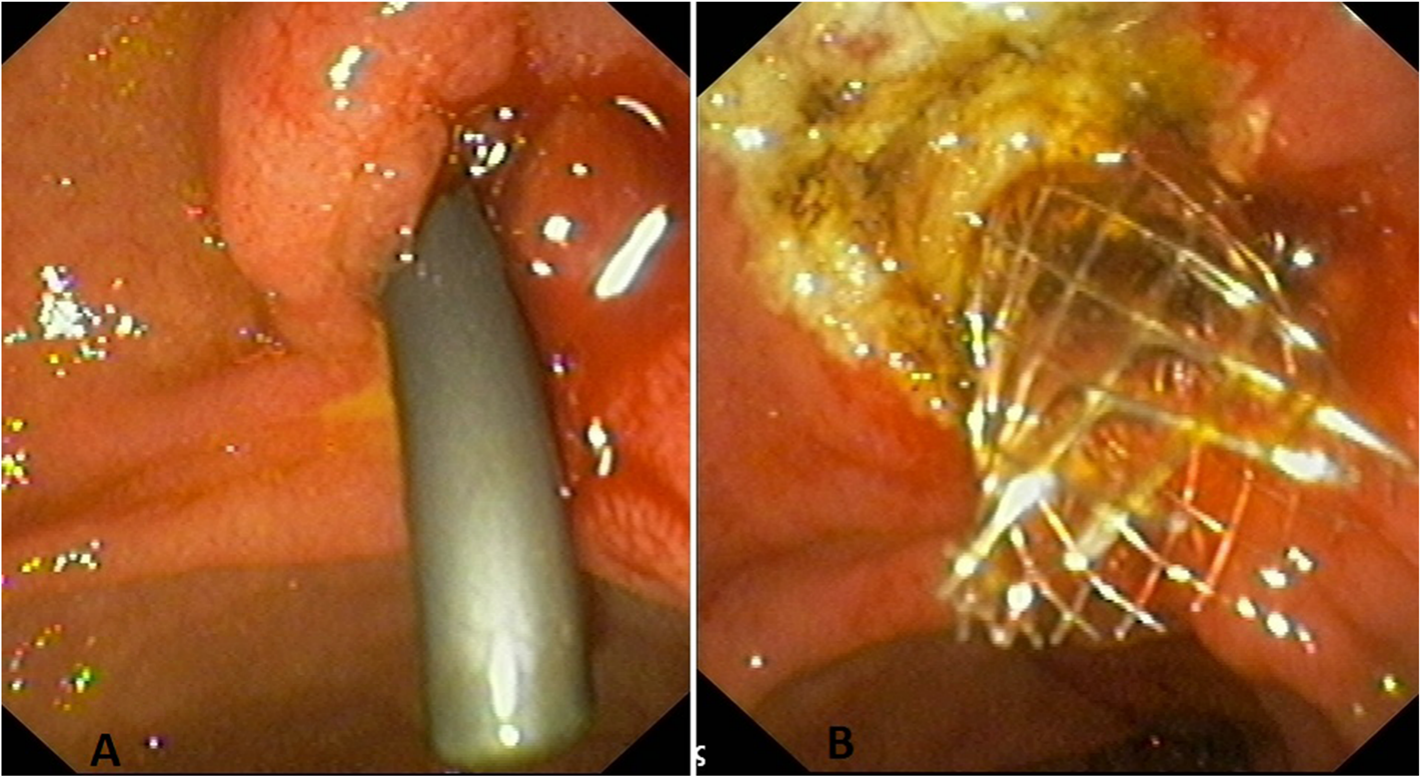
**(A) Image of the ampulla after ampullectomy, endoscopic sphincterotomy and the placement of a plastic 10 Fr biliary stent.** Post ampullectomy endoscopy **(B)** The papilla after additional excision of remnant tissue in combination with argon plasma coagulation and placement of a partially covered metallic biliary stent, 1 month after the initial intervention. One month later, additional argon plasma coagulation and metallic stent placement.

A 77-year-old male presented with painless obstructive jaundice. He had a history of right nephrectomy for a T_2_N_0_M_0_ renal clear cell carcinoma 3 years ago. Ultrasound and abdominal computed tomography (CT) scanning depicted the common bile duct dilated up to its distal end. Endoscopic retrograde cholangiopancreatography (ERCP) revealed an ampullary tumor (Figure 1). Consequently, ampullectomy with endoscopic sphincterotomy and placement of a plastic 10 Fr biliary stent were performed (Figure 2A).

**Figure 3:**
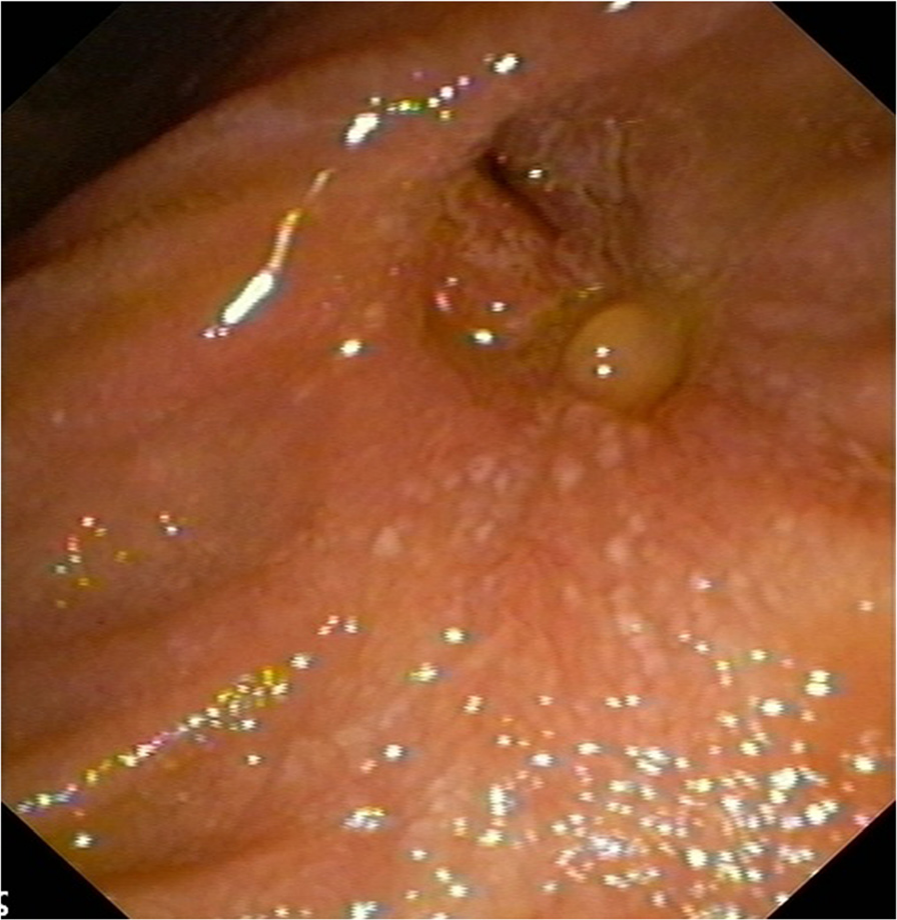
Endoscopic image of the papilla 6 months after treatment, depicting good local control of the lesion.

Histology showed a clear cell carcinoma, consistent with renal origin. Immunochemistry confirmed the diagnosis [vimentin(+), CD10(+), CK8(+), RCCa antigen(+)]. Further evaluation with chest CT and radionuclide bone scanning revealed the presence of lung and bone metastases. The presence of multiple metastatic foci excluded the need for pancreatoduodenectomy and the patient was treated with oral sunitinib. For better palliation, repeat ERCP was performed a month later and additional excision of remnant tissue was performed in combination with argon plasma coagulation (APC) and placement of a partially covered metallic biliary stent (Wallstent; Boston Scientific, Natick, MA) (Figure 2B). Six months later the stent was removed and multiple biopsies showed no evidence of residual tumor (Figure 3). The patient was re-evaluated with endoscopy every 6 months. The patient succumbed to metastatic disease 1.5 years later without jaundice or abnormal liver function tests.

## Conclusion

Renal cancer counts approximately for 3.8% of all adult malignancies [1]. The treatment for localized disease is radical nephrectomy, despite the fact that recent data suggest that less extended procedures in selected patients, such as nephron-sparing surgery (partial nephrectomy) as well as laparoscopic procedures, hold the same results in terms of survival rate [2-6]. Surgical excision is considered curative in 71 to 97% of patients with localized disease (pathologic stage pT1-2), whereas 5-year cancer-specific survival rates after nephrectomy decrease to 20 to 53% for patients with locally advanced tumors and below 15% for patients with metastatic disease [7]. Respectively, the rate of recurrence, even in cases of resection with curative intent, is high, ranging from 20 to 30%. It is estimated that, in total, 50% of the patients with renal carcinoma will present with or eventually develop metastatic disease [8]. Adjuvant therapy consisting of IL-2 and IFN-a, which was considered the standard of care for many years, held very low response rates. Recent advances in our understanding of the biology of renal cell carcinoma led to the development of novel targeted therapies such as mTOR (mammalian target of rapamycin) inhibitors as temsirolimus or the inhibitors of the split-kinase-domain family of receptors of tyrosine kinase sunitinib and sorafenib, which prevent tumor angiogenesis through vascular endothelial growth factor inhibition (mTOR and VEGF are commonly used and may be explained but not substituted). Response rates are currently under investigation in trials; sunitinib, however, has been established as first-line treatment for advanced renal cell carcinoma (RCC) [9]. Usual sites of metastatic spread are the liver, the lungs, the brain and the bones, whereas less common sites are the gallbladder and the urinary bladder. Metastases usually occur in the first 3 years after nephrectomy. However, metastatic foci from renal cancer have been reported as late as 25 years after radical nephrectomy which was considered curative [10].

Ampullary metastases from RCC are quite rare. Sporadic reports of isolated metastases in the ampulla of Vater involved lesions which presented with obstructive jaundice [11], malabsorption [12] or obscure gastrointestinal bleeding, with the prevailing mode of spread being the hematogenous route [13]. Diagnosis is usually set by endoscopic biopsy, since cross-sectional imaging may often fail to delineate a lesion. The treatment proposed for isolated lesions is pancreatoduodenectomy, since data demonstrate sufficient median survival (26 months) and an actuarial 5-year survival of 75%. It is important to denote that, out of the tumors that metastasize in the pancreas and the periampullary region, renal cancer yields the most favorable prognosis; therefore surgical excision is advocated in cases of isolated foci [14-16]. However, this operative approach is not indicated in the presence of multiple metastatic foci and such patients are treated with systematic chemotherapy. The therapeutic challenge is the presence of subsequent complications due to the presence of an ampullary lesion such as in our case, in which intervention was mandatory for the relief of obstructive jaundice. The efficacy of endoscopic resection for benign lesions of the ampulla has been proven [17,18] and has been advocated for small tumors in high-risk patients as a low-risk, minimally invasive procedure [19]. Tumor-free margin can be obtained despite the fact that there had been past reports of local recurrence up to 26% when endoscopic snare excision was utilized [20], since techniques such as APC mitigate such concerns [21]. It is clear that limitations to the efficacy of the method, such as the size of the lesion (larger than 50 mm) and the imaging features in the endoscopic ultrasound or intraductal ultrasound [22], do not apply strictly when the procedure is palliative, such as in our case. It is evident that the ideal result would be ampullectomy with clear resection margins, but the aid of fulgurating techniques such as APC may provide equal results. In our patient, local control was achieved until he succumbed. It seems that in the hands of an experienced endoscopist, endoscopic ampullectomy is a safe and effective procedure in order to provide palliation from metastatic foci in patients with disseminated disease, as well as in high-risk patients who are not candidates for pancreatoduodenectomy.

Keypoints for successful outcome are negative resection margins and endoscopic surveillance [23], but primarily clinical suspicion that the presence of painless jaundice in a patient with history of renal cancer and negative CT scanning for pancreatic or other causes of obstruction should alert for prompt investigation for an ampullary metastasis.

## Consent

Written informed consent was obtained from the next of kin of the patient for publication of this Case report and any accompanying images. A copy of the written consent is available for review by the Editor-in-Chief of this journal.

## Abbreviations

APC: Argon plasma coagulation; CT: Computed tomography; ERCP: Endoscopic retrograde cholangiopancreatography; IFN: Interferon; IL: Interleukin; RCC: Renal cell carcinoma.

## Competing interests

The authors declare that they have no competing interests.

## Author's contributions

AV and AP performed endoscopy and reviewed the images. EK collected the data. AK, CS and GPF reviewed literature,drafted and wrote the manuscript. All authors read and approved the final manuscript.
